# Mapping communities as complex adaptive systems: A study of the response to violence against women by communities in Samoa

**DOI:** 10.1371/journal.pone.0290898

**Published:** 2023-10-19

**Authors:** Hattie Lowe, Louisa Apelu, Laura Brown, Helen Tanielu, Jenevieve Mannell

**Affiliations:** 1 Institute for Global Health, University College London, London, United Kingdom; 2 Samoa Spotlight Initiative, United National Development Programme, Apia, Samoa; 3 Centre for Samoan Studies, National University of Samoa, Apia, Samoa; LSHTM: London School of Hygiene & Tropical Medicine, UNITED KINGDOM

## Abstract

This paper explores the concept of communities as complex adaptive systems in the context of violence against women (VAW) prevention. Using thematic network analysis on data from 80 semi-structured interviews with community members in Samoa, we found that communities exhibit many properties of complex adaptive systems. Within nested systems, diverse and dynamic agents interact based on their knowledge and attitudes, which changes over time, leading to emergent and unpredictable outcomes. The functioning of communities and their response to VAW is a product of non-linear and emerging relationships and interactions between systems components at the community level. The approach we propose for conceptualising communities as complex adaptive systems provides a structured method for designing and evaluating community-based interventions that are grounded in the local context and existing resources. With in-depth knowledge of how a community works, interventions can be better equipped to address wicked problems such as VAW.

## Introduction

Violence against women (VAW) is a ‘wicked’ problem, driven by a myriad of intersecting social, environmental and political factors [1]. Structural factors, such as colonialism, patriarchy and climate change [2], interact with community factors such as social norms [3], to create environments that enable and sustain VAW. How VAW manifests is highly context-dependent [4], and is driven by clusters of different risk factors that interact and exert influence on one another [2]. As such, VAW is a prime example of a complex social problem that requires nuanced and adapted interventions to address it.

Targeting drivers of VAW at the community level has been a central focus of many recent VAW prevention interventions, with promising evidence of effectiveness, particularly in addressing the harmful social norms that perpetuate VAW in communities in low- and middle-income countries (LMICs) [3, 5]. To advance understandings of how community-based interventions work to prevent VAW, this paper draws on systems thinking to conceptualise communities in which VAW interventions are implemented as complex adaptive systems (CAS). This follows a move towards locating complexity within the systems in which interventions are implemented, rather than viewing complexity as an inherent property of the intervention itself, i.e. multi-component VAW prevention interventions are often described as complex [6, 7]. Thus, VAW prevention interventions are conceptualised as events within a system that attempt to disrupt the system’s functioning to bring about change. This moves beyond the traditional linear model of intervention cause and effect to evaluate how diverse and moving parts within a complex system interact with each other, the intervention components, and the context, to achieve the desired outcomes [6]. By framing community-based VAW prevention interventions as events within systems, we can begin to see how an intervention might disrupt the current system which perpetuates violence, to bring about change in preventing it [8].

Systems thinking has been applied to healthcare systems and their responses to VAW [9, 10], as well as interventions implemented in school settings with adolescents [11]. However, despite the promise of systems thinking in intervention science and the growing number of VAW prevention interventions being implemented at the community level, to the best of our knowledge, systems thinking has not yet been applied to communities in the context of VAW prevention. This has resulted in a lack of tools to create comprehensive system-wide solutions to VAW at the community level, while also failing to put communities at the centre of knowledge production for designing local solutions [12]. Towards this aim, this study analyses qualitative data collected as part of the EVE Project in Samoa [13], locally known as *E le Sauā le Alofa* (Love Shouldn’t Hurt), on how communities respond to VAW. The analysis has two aims: 1) to examine the community response to VAW in Samoa and 2), to develop an approach for applying CAS theory in the design and evaluation of community-based interventions.

### Communities in Samoa

The social structure of Samoan communities provides an illustrative case study for mapping communities as CAS. A small independent state in Polynesia (central South Pacific Ocean), Samoa has a size of 2,831km^2^ and a population of approximately 200,000 inhabitants across two main islands [14].

Samoan society dates back more than 3,000 years, with an indigenous culture guided by the *fa’a Samoa* (the Samoan way) and practice of a complex polytheistic religion. Today, Samoan society has been transformed to varying degrees by powerful external forces, such as colonialism and Christianity [15]. The *nu’u* (village), *āiga* (family) and *fono* (chiefly council) are foundational to community life. Approximately 70% of Samoans reside in villages, typically comprising between five and fifteen *āiga potopoto* (extended families which can be made up of more than 15 individuals living on the same area of land), or more in larger villages, who have strong genealogical ties to the village and its customary land [16]. The majority of villages are governed locally by the *fono* which is comprised of *matai* (chiefs), who are the heads of family and represent their families’ interests in the *fono*. Men and women in Samoa have equal rights to own land and acquire chiefly titles, however, *matai* titles are usually bestowed to men, which is a respected norm. While Samoa has a parliamentary democracy, the constitution also allows for locally defined Samoan customs. The 1990 Village Fono Act gives some authority to the *fono* to define local by-laws, permitting they are not in contradiction to State laws [17]. The *fono* usually meet once a month to make decisions about the management of the village, such as the enforcement of locally defined by-laws, with the aim of maintaining peace and social cohesion. Religion is also an integral component of social identity. Since widespread conversion of Samoans to Christianity after introduction by 19^th^ century missionaries, Christian values have played an important role in (re)defining social roles and activities [17]. Approximately 97% of Samoans identify as Christian [18]. Evening prayers and Sunday services are mandatory for most Samoans, and conservative and patriarchal ideals of monogamy and women’s service to their husbands have eroded traditional marital practices and redefined familial relations [17].

Women in Samoa experience high rates of violence, with population level data estimating that 39.6% of ever-partnered women have experienced physical, sexual or emotional violence from an intimate partner in their lifetime, and 31.5% in the past 12 months [19]. The advancement of gender equality in Samoa has progressed in the last 30 years, with national efforts leading to increased numbers of women holding *matai* titles, parliament seats, paid jobs, and managerial and leadership roles [20]. Despite this progress, women still generally hold lower status in social, economic and political life, which is characterised by hierarchical male authority. This is in contradiction with some aspects of the indigenous culture, in which women were afforded powerful statuses as *feagaiga* (sisters) and *tamasa* (sacred offspring) [21, 22]. An estimated 22% of *matai* are female (an increase by around 7% in the last 5 years), but in many villages, it is not unusual for female *matai* to be excluded from monthly *fono* meetings [14, 20, 23]. This exclusion has been justified by the application of the concept of *o le va tapuia* (the sacred space), which outlines a covenant of respect between brothers and sisters. Within this, it would not be appropriate for female *matai* to attend meetings of the *fono*, where men like to ‘jest’ with each other around aspects of sexuality [23]. As only those with a *matai* title can be elected to national parliament, this exclusion of women from local decision-making reproduces the broader limited female political representation in the Samoan government, which despite progress in the last 30 years, still falls behind levels of female political representation in other Pacific nations [20]. Village level *komiti* (women’s committees), traditionally responsible for promoting community health and hygiene, are under the governance of the village *fono*, who are typically men [23]. Similarly, *Sui o Tamaitai* (women’s village representatives) appointed by the *komiti*, and organised under the Ministry for Women, Communities and Social Development, have been active since 2004 as liaison between the *komiti* and the government, but also have limited opportunity for participation in decision-making in the *fono* [23]. Identity within the family can also contribute to women’s social status, a particularly salient example being that of *nofotane* women. *Nofotane* women, who reside in their husband’s village after marriage, are afforded a different social role within the *āiga* and the *nu’u*. They are expected to serve their husband’s family and often experience high rates of violence. These social structures and interpretations of Christianity are considered as foundational to the widespread perpetration of male VAW in Samoa [24].

## Methods

### Theoretical framework

In this study, we apply systems thinking to define communities in Samoa as CAS, drawing upon the work of numerous scholars [25–30]. In **Table 1**, we present definitions of key characteristics of CAS. To define community in this study, we use a place-based definition, in which communities comprise groups of individuals who are linked geographically and socially to a location [31]. This definition is appropriate for the context of Samoa when applied to villages. As described above, traditional villages typically comprise several extended families with strong ties to the land and each other.

**Table 1:** Definitions of key characteristics of CAS.

Characteristic	Definition
Adaptation	System behaviour changes in response to an internal or external input [30].
Nested systems structure	Complex, adaptive systems are nested within larger supra systems, and also have smaller sub-systems embedded within them [26]. These systems are interconnected and influence each other across levels.
Interaction between dynamic agents	Complex adaptive systems are made up of diverse and dynamic agents that act based upon their knowledge, experiences and the local context and its formal and informal rules [26]. Complex, adaptive systems are characterised by the interaction between these agents, rather than the individual agents themselves [25].
Context dependency	The actions of, and interactions between, agents within complex adaptive systems depend heavily on the context, including the available resources, sociocultural perspectives, rules and histories [26].
Feedback loops	Feedback loops appear in complex adaptive systems when the outputs of a system feedback and contribute as inputs into the same system. Reinforcing feedback loops increase the rate of change of one factor in a system in one extreme direction and can result in ‘vicious cycles’ when they reinforce negative factors, whereas balancing feedback loops reverse the direction of change as a result of a change in the system [27, 29, 32].
Emergence	Complex adaptive systems display emergent behaviours, including the ‘spontaneous creation of order’ whereby individuals or smaller systems come together and self-organise as a collective [27]. These emergent behaviours are properties of the system as a whole, rather than properties of the individual parts [30].
Unpredictability	Actions and outcomes in complex adaptive systems are often unpredictable because agents act and interact in non-linear, interconnected ways, and the action of one agent can alter the action of another [28, 30].

### Study design

The data presented in this paper come from the *E le Sauā le Alofa* project, a participatory research project working to co-develop a VAW prevention intervention with ten Samoan communities [13]. We present qualitative data from individual semi-structured interviews with village representatives and peer-led semi-structured interviews with community members.

### Recruitment

Twenty village representatives were purposefully selected from the existing network of the local partner organisation: the Samoa Victim Support Group (SVSG). Selection was based upon representing urban/rural location, size, and cases of VAW reported to SVSG in the last 15 years. One man and one woman were selected from each village in October 2020. In November 2020, village representatives attended a three-day workshop with comprehensive training on qualitative interviewing led by HT at the National University of Samoa (NUS). Village representatives each recruited three individuals from their community to participate in the peer-led semi-structured interviews. Village representatives were encouraged to recruit a diverse group of participants to bring different perspectives to the research. They approached potential participants face-to-face and recruited village leaders, religious leaders, members of the women’s committee, individuals without a specific title, and survivors and perpetrators of violence.

### Data collection

Semi-structured interviews took place between November 2020 and April 2021, first conducted by SVSG with the village representatives as participants during a training workshop (n = 20), and then by the village representatives with members of their community in a community setting, using the same topic guide (n = 60). The topic guide included questions around the types, causes and impact of VAW in their villages, as well as local prevention strategies. Small modifications were made to the topic guide after the first round of interviewing. All interviews were conducted in Samoan, lasting between 30 and 60 minutes. They were audio recorded on mobile phones and then transcribed and translated into English by SVSG. Transcripts were anonymised and only the study team (HL, JM and LB) and SVSG staff had access to a secure file with participant information.

### Data analysis

Data analysis took place across two stages. Firstly, anonymised interview transcripts were imported into NVIVO12 where thematic network analysis was conducted by HL, JM, and CV. Thematic network analysis is a rigorous method for conducting thematic analysis, facilitating the structuring of data across different levels of themes [33]. Researchers read the transcripts multiple times before applying an initial set of inductive codes, guided by two broad categories: 1) factors that drive VAW and 2) local responses to prevent VAW. These codes were grouped into higher order themes that described the mechanism of how communities respond to VAW, for example ‘reporting cases to local leaders’. These descriptive themes were grouped into global themes, summarising responses to VAW at a more theoretical level, bringing together numerous descriptive themes, for example ‘local ownership of the VAW response’. As a final step, researchers created a visual map in NVIVO12 to facilitate discussion of the thematic network map between the research team and SVSG. SVSG provided feedback leading to modifications of the analysis map, ensuring it was grounded in local knowledge.

Secondly, HL extracted the global themes related to the local response to VAW produced during the first stage of analysis, with their subsequent codes and quotes, into a spreadsheet. To begin developing an understanding of the system in which the VAW response was taking place [34], a causal loop diagram was created by reading through the data under each of the global themes and mapping out by hand the response that community members were describing. As the diagram expanded, we began to organise the actions taking place within the system in response to VAW across different domains of the system itself (individual, family, neighbourhood, village). This mapping included visualising the community response pathways and reinforcing feedback loops. Following this, a second round of deductive coding took place, using a pre-defined coding framework which was based upon key characteristics of CAS from a review of existing literature (Table 1) and the causal loop diagram as a visual aid representing the complexity within the system [27]. The aim of this round of coding was to apply concepts of CAS to the community response to VAW in Samoa.

Ethical approval for this study was granted by the research ethics committees of University College London (ref: 9663/002) and the National University of Samoa (ref: 20200609). As well as adhering to SVSG safeguarding procedures, local ethical guidelines were co-produced with village representatives before data collection began and were adhered to throughout. All participants who took part in the interviews provided written informed consent to participate, be audio recorded, and for their anonymised data to be used in research outputs.

## Results

The response to VAW in communities in this study is characterised by non-linearity, unpredictability, context dependence, nested systems and the diverse agents and their interactions with each other and the environment. We present a map of the community response to VAW in Samoa, followed by the application of a CAS approach to Samoan communities.

### Mapping the community response to VAW in Samoa

**Fig 1:**
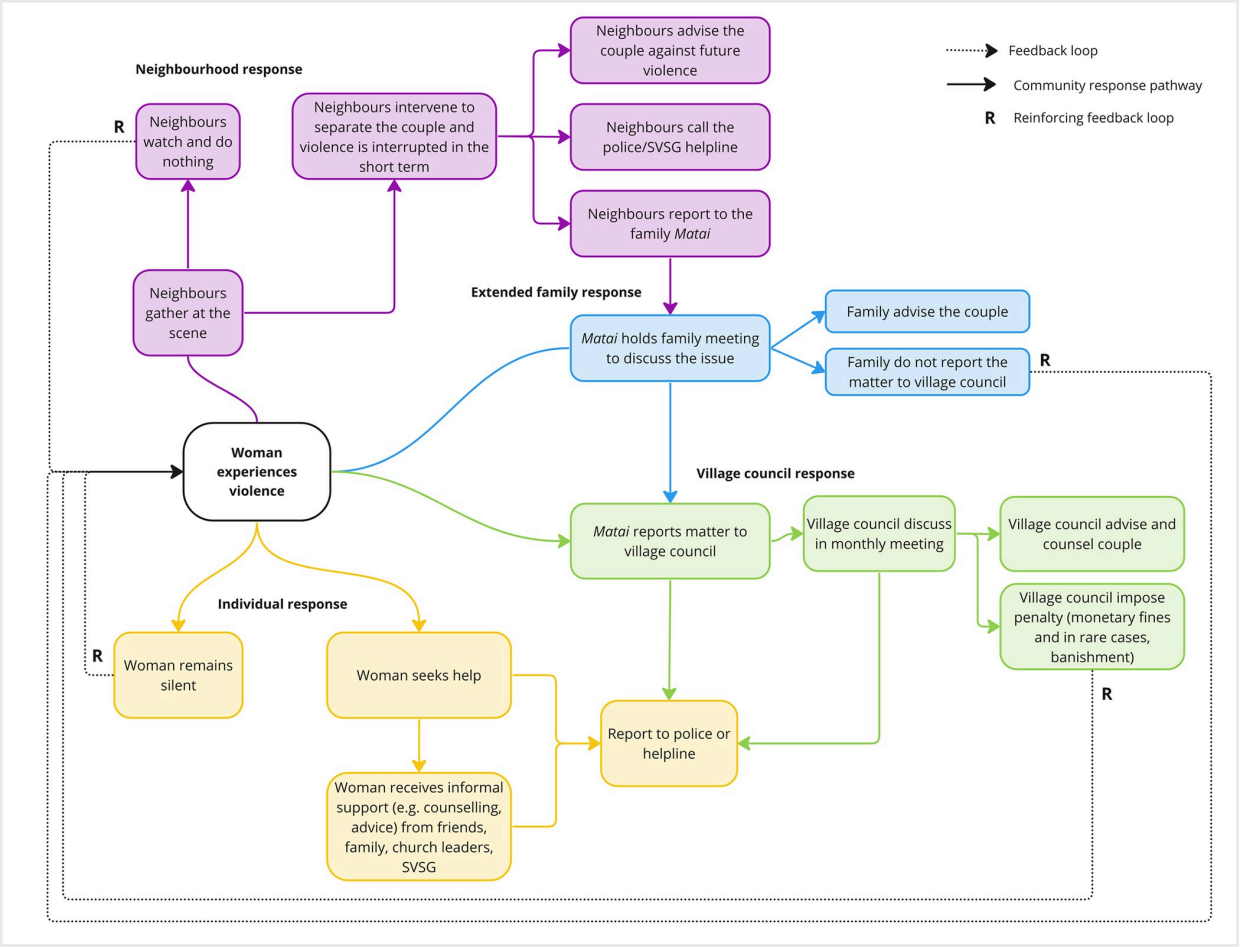
Causal loop diagram of the community response to VAW in Samoa across individual, neighbourhood, extended family and village council domains.

When a woman experiences violence in Samoa, participants described a response that took place across numerous sub-systems: the individual woman and her close social network, the neighbourhood, her extended family, and the *fono* (**Fig 1**). The ways in which these systems respond are diverse and unpredictable, dependent on many different contextual factors such as formal and informal system rules and a communities’ collective history. We present a summary of the non-linear pathways through which survivors seek and receive support, perpetrators are punished, and violence is prevented or perpetuated.

Community members described that often when women experience violence, they remain silent for reasons including pervasive social norms blaming women for violence and ascribing violence as a private family matter, fear of future violence, and to protect their family’s reputation. If violence is seen or heard in the neighbourhood, community members described people gathering at the scene, sometimes intervening to separate the couple, other times just watching the scene unfold, not wanting to get involved in other people’s business. If the couple’s extended family witness or hear about the violence, they might hold a family meeting to discuss the issue, or report the matter to the *fono*. Community members described that many families don’t report violence to the *fono* to protect their honour and avoid being fined in accordance with local by-laws which state physical violence is punishable by fines. If violence is reported to the *fono*, a warning and informal support (advice and counselling) is typically provided in the first instance. This depends on how severe the incident was, and if it was the first occurrence. Cases in which the woman was badly injured, or the family have been warned before, were described as more likely to result in the imposition of a fine. Community members described when severe cases cannot be resolved at the village level, they are usually reported to other authorities such as the police.

### Characterising communities in Samoa as complex adaptive systems

Mapping the response to VAW in Samoan communities enables us to explore the ways in which communities can be characterised as CAS, which we describe below.

#### Nested systems structure

The issue of VAW in the communities in this study exists within a nested systems structure, an important characteristic of a CAS. Communities in this study (in this case, the ten villages participating in the *E le Sauā le Alofa* project) are nested within larger supra-systems which include the church, the state and the *Fa’a Samoa*. Within communities exist nested sub-systems, including the family and the *fono*. These systems are interlinked and influenced by each other, all contributing to the local VAW response. There are contextual reasons as to why the issue may be passed from one system to another, such as when the family *matai* reports a case to the *fono* (e.g. hierarchies and local rules) and also why the issue is kept within systems (e.g. social norms or anticipated negative repercussions), as described by this participant:

“*I have come to learn that the village tried to hide or keep things hush hush when a woman is experiencing violence from a partner or husband*. *I think it relates to how they want to maintain a good image of the village and secondly*, *the families do not want to be punished by the village council…if I report my son in law to the village council for abusing my daughter*, *it will add more to the problem because then my family will argue over who will finance the penalty handed down by the village council*.*”* (Woman, *nofotane*/wife of high chief, village 2)

In the local response to VAW, participants described the *fono* as the highest level of authority with the power to punish perpetrators. In rarer cases, they described violence being reported to the police.

Male authority appeared to pervade the local response to VAW, which is evident at all levels of the nested sub-systems and supra-systems structure. *Matai* (who are typically male) make family decisions regarding how cases of VAW in the family will be dealt with, and *matai* sit on the *fono* to make community decisions about if and how to punish male perpetrators:

“*It’s everyone’s responsibility*, *not just the village council or the high chiefs*, *because the village council is mostly made up of men*.*”* (Man, high chief, village 7)

Male-dominated family and council structures at the village level are characteristic of the male dominated church and state systems at the structural level. If women seek support from the church when experiencing violence, they are typically counselled by a male minister/pastor, as there are few female religious leaders in Samoa. Despite that in some denominations the wives of pastors are involved in resolving issues pertaining to families, no participants mentioned that women could be counselled by women in the church.

While there are a small number of female *matai*, they rarely attend *fono* meetings because of the sacred covenant between brothers and sisters, meaning that women village leaders would rarely be involved in deciding how perpetrators should be punished. Other sub-systems exist within communities, such as women’s committees, however, these sub-systems led by women seemed to have little influence over community-level decisions such as local law enforcement and how male perpetrators of VAW should be dealt with.

#### Interaction between diverse and dynamic agents

A diverse group of individual agents are involved in the community VAW response described by participants, acting based on their knowledge and awareness, local context, and formal and informal system rules. Women, neighbours, families, the village council, religious leaders, local non-governmental organisation (NGO) representatives and the police, situated across the various nested systems, react and interact with each other. Their actions are heavily influenced by their environment, for example the informal system rules such as the social norms that blame women for violence, and the formal system rules, such as the local by-laws that make physical violence punishable by fines. The actions of one agent cannot be viewed in isolation because it is the interactions between agents that characterise the CAS.

The interaction between community members and the *fono* illustrates the importance of interactions between agents. *Matai*, selected by families to sit on the *fono* with powerful and influential positions within their community, are responsible for local governance and the maintenance of peace. Community members see them as role models, however, some participants in this study described their leaders as also being perpetrators of VAW. Consequently, they felt less motivated to prevent VAW and support survivors because their leaders were not setting a good example in their own families:

“*As leaders of families*, *the [village council] should set examples to the rest of the village on how to lead their families in peace*, *not using their fists…But you see*, *that is the problem*, *some of those sitting at the village council are perpetrators of violence themselves*.*”* (Man, untitled, village 3)

Agents in the system are dynamic and constantly changing. Not only is there mobility of agents in and out of communities, but the same agents change over time. An example of how agents have changed over time is the shifting attitudes towards and greater awareness of VAW and its consequences in Samoa due to external factors such as media coverage and awareness raising workshops. These external inputs instigate a diffusion of new social norms around the unacceptability of VAW throughout communities. Community members related this to people in their villages (agents) challenging the informal system rules (social norms) that support the use of VAW, altering the interactions between agents. Because of the greater awareness and growing unacceptability of VAW, community members thought that neighbours might be more likely to intervene when they see or hear VAW taking place. One participant described how this new knowledge benefited her own relationship:

“*There has always been violence in our community*, *but with awareness programs that have visited our village over the years*, *I can see positive changes*… *I used to think that since my husband is the head of my family*, *whatever he says goes*, *and as a wife*, *I should always abide by it so that my husband will not get angry and lash out at me either verbally or physically*. *But with an increased understanding of the consequences of VAW from what I’ve seen and heard*, *I’ve gathered the courage to confront my husband when I think what he wants is not right*. *At first*, *he was shocked that I could talk back at him*, *but eventually*, *he came around*, *and discussed things in a peaceful manner with me*, *which to me*, *is a small change*, *but a change worth celebrating*.*”* (Woman, high chief, village 2)

#### Feedback loops

There are numerous feedback loops in the response to VAW in Samoan communities, occurring when the outcome of one pathway feeds into the issue of VAW as an input. These feedback loops are reinforcing and create a cycle of VAW perpetuation, driven largely by social norms and other contextual factors. As described above, women may choose to remain silent about the violence they experience for a number of reasons, including fear of being blamed and the shame it would bring to their family:

“*…they will put up with the violent treatment instead of seeking help*. *This is especially the case for unemployed women who depend on their husbands for a living*.*”* (Woman, village representative, village 3)

“*…she does not want to tell others about the violence she is experiencing because she is ashamed that people might gossip about her and her family*. *She is more or less protecting the name of her family while she suffers in silence*.*”* (Man, talking chief, village 1)

Remaining silent means that women don’t receive help or support to stop the violence from happening again, and so the cycle of violence is reinforced, as shown by the dotted lines and the R symbol in Fig 1. Similarly, when neighbours, friends and families do nothing to intervene or report VAW, the cycle of VAW is again reinforced.

#### Emergence and adaptation

The communities in this study displayed emergent behaviours in their response to VAW, which is constantly evolving as a result of internal and external inputs. There are examples of spontaneous self-organisation in communities, in which external inputs instigate community members to join together to collectively tackle VAW. For example, widespread media coverage of cases of VAW was described by community members as creating momentum to tackle the problem. Community members described feeling ashamed when reports of VAW come from their own villages, and sadness when they hear stories of women who have been injured or killed from other villages. This seemed to result in a strong determination to work together and take ownership to prevent VAW and support survivors by speaking out and reporting:

“*Before*, *people just minded their own business when a woman is experiencing violence next door*. *Nowadays*, *with the increased public awareness on violence*, *people are making it their responsibilities to help out*.*”* (Man, talking chief, village 1)

“*I think violence against women can be prevented in our village if everyone*, *I mean everyone*, *including the children*, *will play their part in being responsible villager… whenever anyone sees or hears of violence against women*, *they should speak up*, *seek assistance*, *tell others so that more people are raising the alert on these issues*, *men will think twice before abusing the women*.*”* (Woman, nofotane/high chief’s wife, village 2)

#### Unpredictability

The response to VAW in the ten communities in this study was unpredictable and the actions of agents did not always produce the expected outcomes. A particular example is how the implementation of local by-laws for physical VAW worked in different ways. While it was expected these by-laws would reduce the prevalence of VAW by deterring perpetrators who would be afraid of the costly fines, which it seemed to do in some communities, in others these by-laws were perceived by community members as preventing physical violence from being reported, or doing little to prevent other types of VAW such as verbal abuse, demonstrating the emergence of unintended outcomes as a result of the actions taken by agents:

“*I have never seen any physical violence against women in my village*, *not for a long time now*. *If it happens*, *just like within my family*, *we tend to hide it from others*. *But being afraid of the traditional fines*, *I hesitate most of the time*, *to hit my wife when I am angry*. *These traditional fines are therefore a deterrent*, *discouraging men from abusing their wives*. *However*, *I can say that emotional abuse and verbal abuse happens every day in every family; the words we say to each other are hurtful and the root cause of anger should be addressed*.*”* (Man, talking chief, village 1)

Whilst fines were seen to be working to deter physical violence, some participants believed the village council’s approach should be expanded further to include additional support for families to prevent violence from occurring in the first place:

“*Having the village council take up the responsibility for punishing the perpetrators of violence is good*, *to stop men from going down that line*. *However*, *I think also that the punishment should include counselling programs for the couple so that whatever causes the violence*, *will be targeted in the counselling program; instead of the village council just handing monetary or banishment fines*.*”* (Woman, wife of high chief, village 5)

#### Context dependency

How the response to VAW played out in the communities was highly dependent on the local context, including available resources, informal and formal system rules, and the geography and history of communities, amongst other factors. Social norms (informal rules) determine how community members act and interact in the VAW response. Formal rules, such as local by-laws and hierarchies, set out the mechanisms through which communities are expected to respond to VAW, such as reporting to the family *Matai* and *fono*. The history and cohesion within villages also influences how agents interact. Communities are diverse, of different sizes, geographies, and histories and have varying levels of social cohesion. In villages with strong leadership, shared history and collective pride about who they are, community members seemed motivated to make changes for the good of their village, including supporting women who experience VAW and reporting perpetrators:

“*We have so much pride*, *that if we nurtured and steered to the right direction*, *we can become a village to be proud of*. *Look around us*, *we can never go without water because the river that runs through the village never runs out*…*we are a very rich village*, *and our people should be proud of that*. *Instead*, *we are known for being so violent*, *oh my gosh*, *I feel like crawling under a rock and hiding every time I hear news of an incident here*. *Our village leaders should help bring this sense of pride back in our villagers… instead of tarnishing the village’s name by committing violence against women*.*”* (Woman, women’s committee leader, village 2)

However, more recently formed and less traditional communities had fewer social ties between families, and these villages appeared to have higher levels of VAW, possibly due to the unwillingness of neighbours to speak up for one another:

“*I think all sorts of violence is happening in my village and I can see that it’s not reducing but increasing*. *This may be because [this] is not your traditional village*, *there are a lot of people from other villages residing here; and some of the people who are banished from their villages for wrongs they have committed*, *ended up settling [here]… this is where I think the problem seems to have worsened*.*”* (Woman, nofotane/wife of high chief, village 10)

## Discussion

Through the analysis of qualitative data on the topic of local strategies for violence prevention, we have shown that the communities in this study can be conceptualised as CAS. They exist in a nested systems structure that is a product of the social and political organisation in this setting, centring around the *village fono* and the *fa’amatai* structures. Within these nested systems are diverse and dynamic agents–community members, leaders and organisations–who interact based on their knowledge and attitudes, the formal and informal rules, and the collective history of the system in which they are situated. These agents are dynamic and evolve over time and this dynamic nature of agents leads to emergent outcomes like spontaneous self-organising in communities to intervene and support women who are experiencing violence. These communities in Samoa also exhibit unpredictability and negative outcomes like the underreporting of VAW which often feedback into the system through feedback loops, reinforcing a cycle of violence. Most importantly, we show that the functioning of these communities and how they respond to VAW is not determined at the individual level, but is a product of the non-linear and emerging relationships and interactions between system components at the community level.

The approach we take for conceptualising communities as CAS makes a valuable contribution to the fields of intervention development research and policy and practice. This is true not only for VAW prevention, but also in other areas of health promotion where communities are increasingly placed at the centre of interventions aiming to improve human health and wellbeing [35]. We discuss the benefits of applying a CAS lens to communities across phases of intervention development and evaluation, using specific examples related to community-based VAW prevention interventions.

During planning and development of a community-based intervention, collecting and analysing formative data with a CAS lens can develop a deeper understanding of the intervention setting. Formative research to understand the intervention context is widely acknowledged as a critical early step in intervention development [36], yet limited practical guidance exists that lays out a step-by-step process for doing so. Our approach to conceptualising communities in Samoa as CAS, which included iterative stages of qualitative data collection, analysis, causal loop mapping and theory building, provides an example through which researchers and practitioners, in collaboration with local stakeholders, can explore how communities are currently functioning, providing a structured and flexible approach that highlights key components of CAS for consideration. These areas should include, but are not limited to, local systems of governance and how communities are situated within broader and narrower social and political structures, who the agents in the system are and how they interact, what processes and pathways already exist in the system for the particular issue under study and if/how they work, and importantly, what the existing system rules and histories are that determine the behaviour and interaction of agents.

With a clear understanding of how a community functions, intervention teams can begin to determine which components of the system the intervention should attempt to disrupt to bring about the desired change as part of the theory building process [30]. Not only does a CAS lens ground intervention theories in the local context for more meaningful and sustainable interventions and outcomes [27], it promotes the exploration of feasible and realistic solutions to context specific problems. These solutions could build on existing resources and pathways that were identified during the formative causal loop diagramming process, as well as attempt to create new ones to address identified problems or reinforcing feedback loops [37]. Our analysis shows that in these communities, there are already mechanisms in place when a woman experiences violence, which appear to have both positive and negative outcomes. For example, the hierarchical organisation of communities in Samoa, centred around the powerful *Matai* and *fono*, is important in the VAW response. An intervention in this setting should aim to leverage the influence of these community structures, for example by training *fono* members to become positive role models and proponents of gender equality in their communities. Similarly, this process has exposed the problematic nature of some of the existing mechanisms, such as monetary fines which seem to be reinforcing rather than preventing VAW, providing an important entry point for disrupting the current functioning of the system to transform reinforcing feedback loops into balancing ones. Conceptualising communities as CAS may also be a valuable approach when planning the adaptation and scaling up of an existing intervention into a new context. It forces intervention teams to explore whether mechanisms from an intervention in one system would manifest the same way in another [26].

The CAS lens may be particularly insightful for the development and evaluation of VAW prevention interventions which focus on gender transformation. Gender transformative interventions attempt to reshape the gender system to be more equitable for the prevention of VAW [38]. Gender norms, informal rules of the gender system, are a critical contextual factor in a CAS that shape the interaction between agents [11]. Before an intervention attempts to shift these often historically entrenched system rules and interactions, it must first understand how these interactions play out in that unique context. In Samoan communities, the gender norms that blame women for the violence they experience prevent women from seeking help. Taking a CAS approach provided us with a structured process for tracing these informal rules and exploring the impact they had on interactions between agents, and on wider system functioning. It also highlighted many potential entry points for attempting to disrupt the system to achieve VAW prevention.

Intervention evaluation also benefits from a CAS lens because it creates space for exploring the unpredictable and emergent outcomes that are typical when a social programme is implemented within a complex system [27]. In our study, community interventions to reduce VAW perpetration by fining perpetrators had variable outcomes. In some instances, it deterred families from reporting cases of violence to the *fono*, perpetuating VAW, and in others, it reduced physical VAW perpetration but enabled other types of VAW, such as emotional and economic abuse, to go unpunished. A systems lens helps to prepare for these unpredictable outcomes in the intervention development phase, as well as trace how and why they manifested during evaluation.

Approaching communities with a CAS lens has numerous benefits for intervention development and evaluation in the field of VAW prevention and wider global health promotion. However, this approach is limited by the differing understandings of what constitutes CAS theory, meaning that application of this theory is not always consistent and comparable [26, 39, 40]. Similarly, use of this theory is still emerging in global health, which meant that in this study, there was limited literature to draw upon in formulating the key components of CAS for our analysis. Nevertheless, using the available literature, we share, to the best of our knowledge, the first analysis to conceptualise communities as CAS in the context of VAW prevention. This can be used as a springboard for developing the concept further. A specific limitation of our study is that we drew upon data from one specific unique context for our mapping. As such, we may have missed important concepts because they were not present in this context, or over emphasised others which are specific to Samoa and might not be relevant elsewhere. While this is a limitation of our study, we believe it provides a strong argument for researchers working on VAW prevention with communities in different settings to apply and develop this conceptualisation further.

## Conclusion

In this paper, we share an approach for conceptualising communities as CAS. We show that the Samoan communities in this study are situated in a nested systems structure and comprise diverse and dynamic agents who change over time. We also show that a community’s functioning is defined by the interactions between agents, which are highly context dependent and often unpredictable. This CAS lens embraces the uniqueness of communities and contexts, while also providing a structured approach through which to gain a deeper understanding of how they function. With in-depth knowledge of how a community works, and realistic targeted approaches that are grounded in the local cultural context and existing resources, community-based interventions can be better equipped to address ‘wicked’ problems such as VAW.

## Supporting information

S1 Checklist COREQ (COnsolidated criteria for REporting Qualitative research) checklist. (PDF)

S1 File (DOCX)

## List of abbreviations

VAW: Violence against women
CAS: Complex adaptive systems
LMIC: Low- and middle-income country
EVE: Evidence for Violence prevention in the Extreme
SVSG: Samoa Victim Support Group
NUS: National University of Samoa
NGO: Non-governmental organisation
